# Toward a Green SPPS: The Use of an Innovative Mesoporous pDVB Support for Environmentally Friendly Solvents

**DOI:** 10.1002/psc.70038

**Published:** 2025-06-30

**Authors:** Luana Lastella, Marco Zecca, Paolo Centomo, Karel Jeřábek, Fernando Formaggio, Ivan Guryanov, Antonio Ricci, Barbara Biondi

**Affiliations:** ^1^ Department of Chemical Sciences University of Padova Padova Italy; ^2^ Institute of Chemical Process Fundamentals of the CAS, v.v.i Prague Czech Republic; ^3^ Padova Unit Institute of Biomolecular Chemistry Padova Italy; ^4^ Institute of Chemistry, St. Petersburg State University St. Petersburg Russia; ^5^ Fresenius Kabi Ipsum Rovigo Italy

## Abstract

This study explores the use of a novel polymeric mesoporous support (pDVB) for solid‐phase peptide synthesis (SPPS), with the aim of improving the efficiency and sustainability of the process. The pDVB support, functionalized with the Fmoc‐Rink amide linker, offers advantages over conventional supports based on gel‐type, lightly crosslinked polymer skeletons, particularly with regard to reduced reliance on swelling capacity, which allows the use of a wider range of solvents. The work focuses on *greener* and eco‐friendly solvents such as TEP, ACN, IPA, and their mixtures with DMSO to replace toxic solvents such as DMF. The synthesis of two model peptide sequences, Fmoc‐LLVF‐NH_2_ and ACP(65–74), showed that pDVB‐Rink performs better than a conventional‐type Rink Amide MBHA support, especially when using environmentally friendly solvents. These results suggest that mesoporous pDVB‐Rink is a promising solid support for SPPS to reduce the use of toxic solvents and to improve sustainability.

## Graphical abstract

A novel polymeric mesoporous support (pDVB) has been proposed with the aim of improving the efficiency and sustainability of SPPS. This new resin enables the use of a wider range of green solvents.

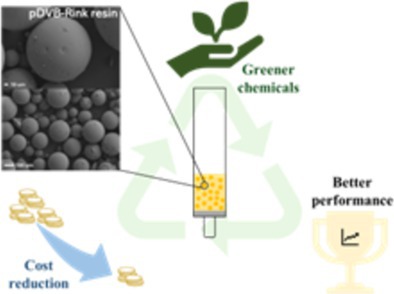

## 1 Introduction

Nowadays, there is an increasing investment in the use of peptides across various fields, including pharmaceuticals, agriculture, food, materials, and diagnostics. Peptides are a valuable class of molecules as they act as intermediates between small molecules and large proteins. They are more selective than small molecules, more stable than proteins, and can penetrate cells more easily than the latter. Additionally, they have lower immunogenicity and offer cost‐effective production. In the pharmaceutical industry, peptides are a key class of active pharmaceutical ingredients (APIs), and in recent years, an increasing number of peptide‐based drugs have been approved. In fact, in 2024, the FDA approved 50 new drugs, including 4 TIDES (2 pepTIDES and 2 oligonucleoTIDES), bringing the total number of peptide‐based drugs available on the market to approximately 120. Many more are in various phases of development, highlighting the growing significance of these products in the pharmaceutical field as well as in diagnostics, drug discovery, and immunology [1]. Peptides and oligonucleotides now account for roughly 10% of all approved drugs [2]. The importance of this class of compounds is also reflected in their market value: In 2023, the peptide market was valued at USD 10 billion, and it is projected to grow considerably to USD 106 billion by 2033 [1], boosted by the approval of GLP‐1 analogs for body weight control [3]. Peptide synthesis is predominantly carried out through solid‐phase peptide synthesis (SPPS), a technique introduced by Merrifield [4] in 1963. Despite numerous advancements over the years in the development of protecting groups, coupling reagents, and optimized reaction conditions, SPPS remains an expensive process. This is primarily due to the large quantities of amino acids and coupling reagents that are required, which necessitate the use of large volumes of solvents to remove the excess materials by filtration and washing steps. Furthermore, the purification steps, which are essential for obtaining the final peptides in high purity, contribute significantly to the overall volume of waste generated. From an industrial perspective, the scale of waste becomes particularly problematic. In large‐scale peptide production, the amount of waste can reach significant levels, with several tons of waste generated per kilogram of API produced [5]. This results not only in economic challenges but also in environmental concerns, especially in relation to the disposal of solvent waste. Another important issue is the toxicity and environmental impact [6] of the solvents typically used in peptide synthesis, such as *N*,*N*‐dimethylformamide (DMF) [7], dichloromethane (DCM) [8], *N*‐methyl‐2‐pyrrolidone (NMP) [9], and *N*,*N*‐dimethylacetamide (DMA) [10]. Many of these solvents are classified as hazardous by regulatory agencies such as the European Chemicals Agency (ECHA), under the Registration, Evaluation, Authorisation, and Restriction of Chemicals (REACH) regulation. Consequently, the need to develop more sustainable and environmentally friendly alternatives is becoming increasingly urgent. This includes adhering to the 12 principles [11] of green chemistry and identifying solvents that are renewable, non‐toxic, non‐volatile, recyclable, biodegradable, and inexpensive. In response to these concerns, the scientific community has made significant strides to enhance the sustainability of the peptide synthesis process [12, 13, 14, 15, 16]. This progress is being driven by two main objectives: (i) replacing toxic reagents and solvents with more environmentally benign alternatives [17, 18, 19, 20] and (ii) reducing the volume of solvent required during the synthetic steps [21, 22]. Numerous studies have been conducted to identify greener solvents that could replace DMF and other commonly used reagents [23, 24]. Initial examples of green solid‐phase peptide synthesis (GSPPS) were extensively documented by MacMillan [25] and by Albericio and coworkers over the past decade, with numerous studies focusing on the use of less toxic solvents such as acetonitrile (ACN) [26, 27], 2‐methyltetrahydrofuran (2‐MeTHF) [28, 29], γ‐valerolactone (GVL) [30, 31, 32], N‐butylpyrrolidone (NBP) [33, 34], *N*‐formylmorpholine (NFM) [35], *N*‐octylpyrrolidone (NOP) [36], propylene carbonate [23, 37], anisole [38], and triethyl phosphate (TEP) [39]. However, some of these solvents come with their own limitations. Moreover, in SPPS, the solvent must effectively support the swelling of the resins, the coupling reactions, the deprotection steps, and the washing processes. For these reasons, finding a single solvent that can perform optimally across all these stages became challenging, with an adequate candidate still not present. To overcome these issues, the research shifted towards the use of solvent mixtures. By combining two different solvents, it is possible to improve the overall chemical and physical properties of the solvent system. Some of the effective mixtures include dimethyl carbonate or diethyl carbonate combined with Cyrene, sulfolane, or anisole [40], and the widely used combinations of ethyl acetate or 1,3‐dioxolane (DOL) with NBP or dimethyl sulfoxide (DMSO) [41]. Although solvent mixtures have addressed some of the synthesis challenges, one key limitation remains: the swelling of the resin. A solvent unsuitable for swelling the typical polystyrene resins leads to a low quality of the final product.

Here, we report the unprecedented use of a new, solid, mesoporous polymer support which can be expanded to a very high extent in many different solvents, with only a little dependence (if any) on their nature. As a result, the morphology of this support under SPPS conditions is essentially unaffected by the nature of solvents used throughout the process of peptide synthesis. The mesoporous polymer (pDVB) is prepared from technical grade divinylbenzene (DVB) and can be easily functionalized using conventional methods. The pDVB resin was used in the form of little spherical beads prepared by suspension polymerization [42]. In this study, we evaluated a selection of solvents recently adopted by the scientific community as more sustainable alternatives. The solvents used were ACN, TEP, DMSO/TEP [43] and DMSO/isopropyl alcohol (IPA). The latter solvent mixture was proposed by Apptec in collaboration with Albericio's group and presented at the 37^th^ European Peptide Symposium. These innovative solvents offer a more sustainable and efficient approach to peptide synthesis, not only reducing the environmental impact but also enhancing the overall efficiency and cost‐effectiveness of the process. Through continued research and development, it is expected that the peptide synthesis industry will gradually move towards greener practices, thus contributing to mitigate the challenges associated with waste generation and solvent toxicity and ultimately fostering a more sustainable future for the pharmaceutical and biotechnological industries.

## 2 Experimental Part

### 2.1 Materials and Methods

*N,N′*‐Diisopropylcarbodiimide (DIC), ethyl cyano(hydroxyimino)acetate (Oxyma Pure), 1‐hydroxybenzotriazole (HOBt), Fmoc‐Rink amide linker, piperidine, and all amino acids (Fmoc‐AA‐OH) used were obtained from Iris Biotech (Marktredwitz, Germany). Rink Amide MBHA resin (loading 0.5 mmol/g) was supplied by CBL (Patras, Greece). DMF, ACN, HPLC‐quality ACN, ethylenediamine (EDA), *N*‐methylmorpholine (NMM), DMSO, diethyl ether, triisopropylsilane (TIS), and trifluoroacetic acid (TFA) were purchased from Merck (Darmstadt, Germany) and TEP from BLD Pharm (Reinbek, Germany). Milli‐Q water was used for HPLC‐MS. HPLC‐MS was performed on a Phenomenex Kinetex XB‐C18 column (4.6 mm × 100 mm, 3.5 μm, 100 Å) with an Agilent Technologies 1260 Infinity II HPLC system and a 6130 quadrupole LC/MS instrument.

### 2.2 pDVB Functionalization

**SCHEME 1:**
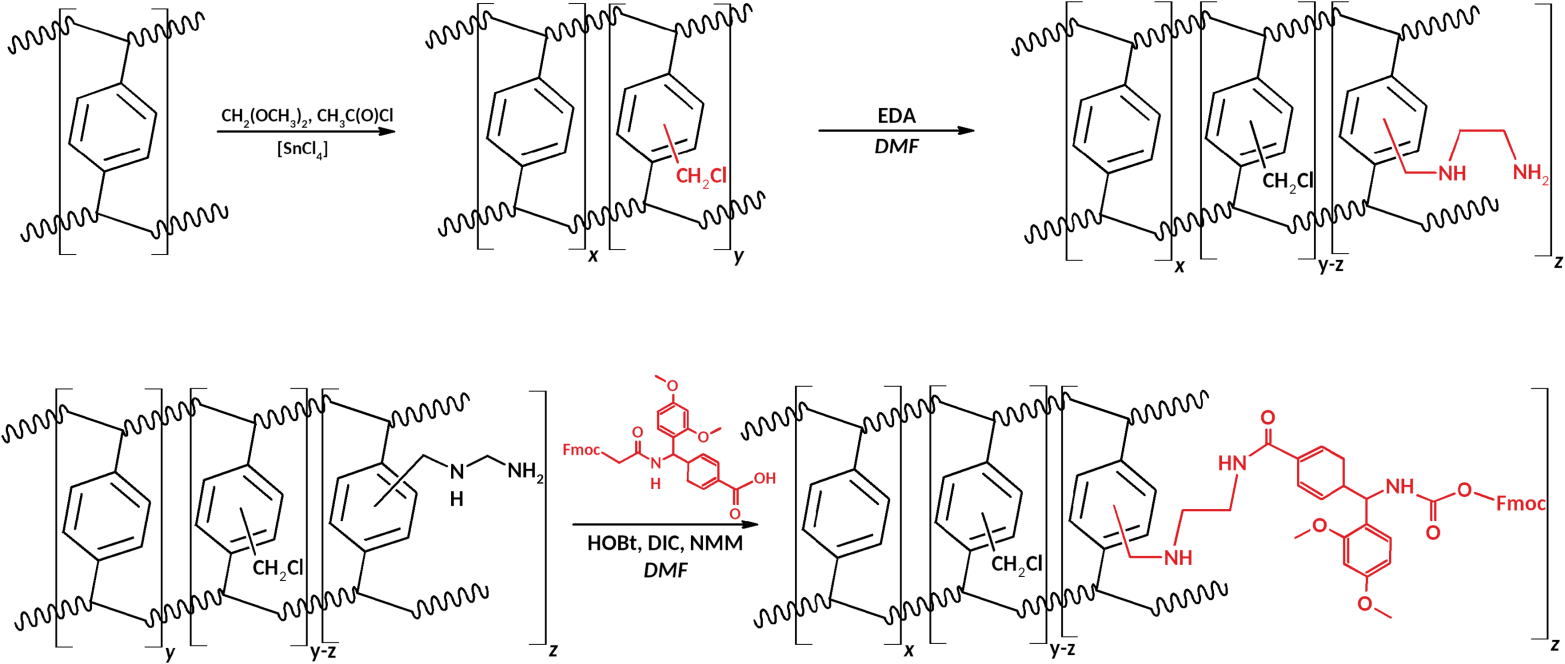
Functionalization of pDVB with Fmoc‐Rink amide‐linker

Before the attachment of the Rink linker, the starting pDVB polymer was chloromethylated using a mixture of dimethoxymethane and acetyl chloride with anhydrous stannic chloride as the catalyst (Scheme 1). The dry chloromethylated resin was swollen in DMF for 30 min. After swelling, the spacer chosen (EDA) was added. Chloromethyl (3 eq per mmol) sites were used, and the reaction was carried out overnight. After washing using DMF, the Fmoc‐Rink amide linker was added. The reaction was allowed to run for 4 h using 2 eq (relative to mmol of Cl) of Fmoc‐Rink amide linker, HOBt, and DIC (1:1:1 ratio) and 2.5 eq of base (NMM). At the end of the reaction, the functionalised resin (pDVB‐Rink) was washed with DMF and DCM, respectively, and dried. The degree of functionalisation was then determined by UV–Vis spectroscopy. The loading obtained was 0.40 mmol/g.

### 2.3 Scanning Electron Microscopy (SEM)

SEM was performed with a Zeiss Sigma HD microscope, equipped with a Schottky FEG source, one detector for backscattered electrons and two detectors for secondary electrons (InLens and Everhart Thornley). The samples were deposited on carbon tape and sputter coated with 15 nm of gold.

### 2.4 Solubility Test

To test the solubility of the Fmoc‐amino acids in IPA and in DMSO/IPA mixtures up to 0.2 M, 1 mL of solution was prepared for each of them in each tested solvent. We were able to solubilize also non‐soluble amino acids by adding the appropriate amounts of OxymaPure and DIC to the 0.2 M mixture, thus yielding the more soluble active ester. The solvents employed were IPA and the binary mixtures DMSO/IPA 25/75 v/v, 15/85 v/v, 10/90 v/v, and 5/95 v/v.

### 2.5 Resin Swelling

The pDVB‐Rink expands upon sorption of liquids: this is referred to as “swelling” here and later, but this expansion is different from the true swelling of the polymer matrix of the lightly cross‐linked gel‐type Rink Amide MBHA resin (see Results and Discussion, § 3.1). In order to assess the swelling behavior of our new resin as compared to that of a standard Rink Amide MBHA resin, we carried out a gravimetric test [43, 44, 45] with the different solvents studied. Two hundred milligrams of each resin was loaded into 6 mL polypropylene syringes equipped with a porous frit. The resins were washed three times with 1 mL of the solvent under scrutiny. Then, 1 mL of the solvent was added, and the solvent–resin mixture was stirred for 4 h at room temperature. Subsequently, the resin was compressed with the syringe plunger to completely remove the solvent. Then, the plunger was slowly retracted to allow the recovery of the swollen resin. The swelling ratio for each resin–solvent combination was calculated using the following equation (*V*_*filter*_ = 0.15 mL; m_resin_ = dry weighed mass of the resin):

$$\text{Degree of Swelling}\left(\mathrm{mL}\cdot g^{-1}\right)=\frac{\left(V_{\text{swollen resin}}-V_{\text{filter}}\right)\left(\mathrm{mL}\right)}{m_{\text{resin}}\left(g\right)}$$

### 2.6 Procedure for SPPS

The SPPS of Fmoc‐LLVF‐NH_2_ and ACP (65–74) was performed for all solvents on the two resins under comparison using a standard Fmoc protocol. The pDVB‐Rink resin has a loading of 0.40 mmol/g while the Rink Amide MBHA resin has a loading of 0.50 mmol/g. Couplings were carried out using Fmoc‐AA‐OH, OxymaPure [46] and DIC, pre‐activating the mixture for 3 min. Then, the mixture was added to the resin and stirred for 50 min. Fmoc deprotection was performed with two treatments (5 min + 15 min) with 20% piperidine in the tested solvent. Washing steps were omitted after coupling but maintained after Fmoc removal (4x1 mL). At the end of each synthesis the peptide was cleaved from the solid support using a mixture of TFA/TIS/H_2_O in a 95/2.5/2.5 ratio. The filtrate was collected and concentrated under a nitrogen flow, and then the peptide was precipitated by addition of diethyl ether. The product obtained was characterized by HPLC‐MS on a Phenomenex Kinetex XB‐C18 column (4.6 mm × 100 mm, 3.5 μm, 100 Å) with an Agilent Technologies 1260 Infinity II HPLC system and a 6130 quadrupole LC/MS instrument.

## 3 Results and Discussion

### 3.1 New Solid Support and Its Properties

Since the beginning of SPPS, low‐crosslinked gel‐type polymers have been predominantly used as supports. In these materials, the accessibility of reaction sites depends on the swelling of the polymer matrix in the reaction environment [47]. Swelling produces a gel in which the polymer backbone acts as a co‐solvent, significantly influencing the nature of the reaction environment [48]. As a result, the synthesis conditions cannot be independent of the polymer. The first generation of polymer supports was based on simple copolymers of styrene and divinylbenzene. However, with such supports, the rate of amino acid incorporation decreases with increasing peptide length [49]. To overcome these issues, several supports with modified chemical structures have been developed [50, 51], particularly incorporating acrylamide or polyethylene glycol moieties (e.g., Champion II, ArgoGel, CLEAR, Tentagel, NovaPeg, or ChemMatrix). Nevertheless, these supports also require swelling, which again limits the choice of suitable solvents. A further improvement could be the use of permanently porous polymeric supports, such as macroreticular copolymers of styrene and divinylbenzene, because they are much less dependent on swelling. However, the pores in these macroreticular resins are formed by void spaces between aggregated polymer nanoparticles [47]. This implies that increasing the pore size enough to meet the steric demands of SPPS unacceptably reduces the wall area where peptide synthesis can be carried out. Consequently, macroreticular resins with sufficiently large pores for SPPS would have limited capacity for synthesis.

In 2009, Feng and coworker described a “solvothermal” polymerization of DVB [52], performed under pressure and at high temperatures in the presence of a tetrahydrofuran‐water mixture. The resulting polymers exhibited unusual properties, such as the ability to apparently swell in n‐heptane, which conventional styrenic polymers cannot do. These polymers have a very high surface area, but in the dry state they apparently have only micro‐ or small mesopores, in the nanometer or lower scale, similarly to the conventional macroreticular polymers of similar surface area. However, their analysis in the solvent‐expanded state [53] using Inverse Steric Exclusion Chromatography (ISEC) [54, 55] shows a very different porosity. Expanded pDVB has extremely high surface area with pores 20–80 nm wide, suggesting that the pore system is foam‐like rather than a network of void spaces between clustered polymer particles. Further investigation [56] on their preparation showed that the creation of this exceptional morphology does not require the exotic “solvothermal” conditions and that the key factors for achieving this foam‐like structure are: (i) a very high content of the cross‐linking component (over 50%), (ii) the use of a porogenic solvent with good affinity for the polymer being generated, and (iii) a very high dilution of the monomers (solvent/monomer ratio of 3 to 10, v/v) in the polymerization mixture. These conditions produce a highly cross‐linked polymer with a rigid backbone and the foam‐like morphology as the result of micro‐syneresis as the main phase separation process during the polymerization [57]. The pores collapse partially when the polymer is dried due to capillary forces, and when the polymer is immersed in a liquid that wets the walls of the pores, they re‐expand. Although the result is apparently the same as the swelling of the polymer framework (increase of the volume upon sorption of a liquid), the underlying phenomenon is different. In spite of this, for the sake of simplicity, the expansion of the pDVB resin has been generically herein referred to as “swelling.” Remarkably, these volume changes are (almost) independent of the nature of the surrounding liquid, meaning that the wet‐state morphology does not depend on the swelling of the polymer matrix itself as critically as in the conventional gel‐type supports.

The pDVB used in this work was obtained as described by Hankova et al. [56] from technical grade DVB (80% pure, with ethylstyrene as the second most abundant component). The basic polymer pDVB was prepared in the form of spherical beads 0.1–0.25 mm and subsequently converted to the pDVB‐Cl as described in Scheme 1. The content of Cl in the polymer just after chloromethylation was typically about 6.9% (1.9 mmol·g^−1^). The reaction of pDVB‐Cl with EDA and the Fmoc‐Rink amide linker allowed obtaining the solid support with a substitution grade of 0.4 mmol·g^−1^.

**FIGURE 1:**
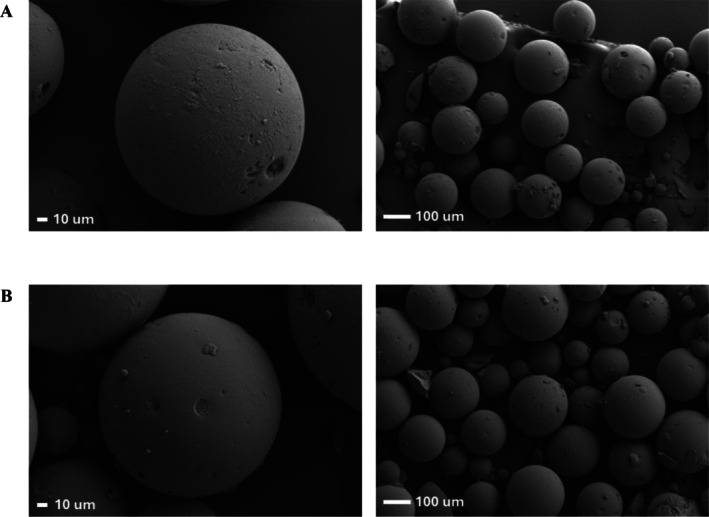
SEM images of pDVB‐Cl (A) and pDVB‐Rink (B).

SEM analyses of chloromethylated pDVB and pDVB‐Rink showed that the polymers were not uniform in bead size in either case, and that functionalization did not affect the material's morphology (Figure 1).

### 3.2 Resin Swelling

**FIGURE 2:**
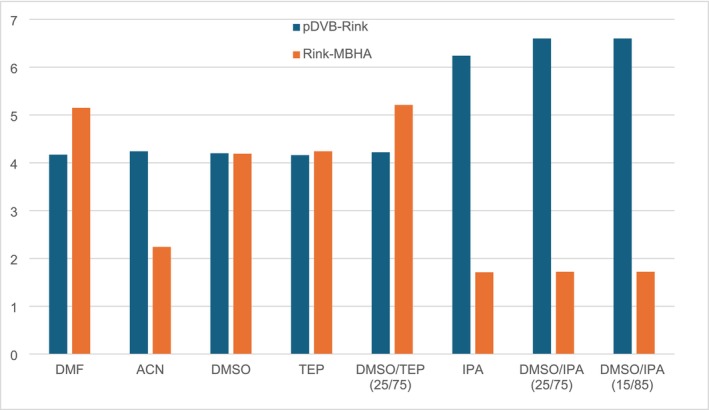
Comparison of the swelling properties of pDVB‐Rink and Fmoc‐Rink MBHA resins in different conditions.

To compare the potential of the new pDVB‐Rink resin as a SPPS support with that of the Fmoc‐Rink MBHA resin, the degree of swelling was evaluated in pure and mixed solvents (Figure 2 and Table 1) [43].

**TABLE 1:** Comparison of the swelling values of the pDVB‐Rink and Fmoc‐Rink MBHA resins in different conditions.

Solvent	Swelling of resin (mL/g)	
	Rink MBHA resin	pDVB‐Rink resin
Loading (mmol/g)	0.50	0.40
DMF	5.2	4.2
ACN	2.2	4.2
DMSO	4.2	4.2
TEP	4.2	4.2
DMSO/TEP (25/75 v/v)	5.2	4.2
IPA	1.7	6.2
DMSO/IPA (25/75 v/v)	1.7	6.6
DMSO/IPA (15/85 v/v)	1.7	6.6

As well known, the swelling properties of the Fmoc‐Rink MBHA resin were strongly dependent on the solvent tested, with a threefold decrease from DMF or DMSO/TEP (5.2 mL/g in both) to IPA and all its mixtures with DMSO (1.7 mL/g in both ratios) and intermediate values in ACN, pure DMSO, and pure TEP. The behavior of the pDVB‐Rink resin was outstandingly different. Its swelling in DMF is only 20% lower in comparison with the Fmoc‐Rink MBHA resin, but remarkably its value in ACN, TEP, DMSO, and DMSO/TEP is the same. Not only this resin was swollen in all solvents and solvent mixtures, but also the degree of swelling was extremely high in those mixtures where the Fmoc‐Rink MBHA resin failed (last three entries of Table 1). Thus, the negligible solvent dependence of the swelling of the pDVB‐Rink resin and its generally high values support the conclusion that with this new support, the range of solvents for the SPPS could be widely extended with a positive impact on the solvent greenness.

### 3.3 Solubility of the Amino Acid Derivatives

In order to evaluate the effectiveness of solvents and mixtures alternative to DMF in peptide synthesis, it is necessary to verify the actual solubility of the Fmoc‐AA‐OH at the concentrations used. Most of the solubility data relevant to this work are already available in the literature [43] for many solvents apart from IPA and DMSO/IPA; hence, they were evaluated only for the latter (Table 2).

**TABLE 2:** Solubility of the different Fmoc‐AA‐OH at 0.2 M concentration in the different conditions studied.

Fmoc‐AA‐OH (0.2 M)	IPA	DMSO/IPA (5/95 v/v)	DMSO/IPA (10/90 v/v)	DMSO/IPA (15/85 v/v)	DMSO/IPA (25/75 v/v)
Fmoc‐Ala‐OH	✔	✔	✔	✔	✔
Fmoc‐Asn(Trt)‐OH	**×**	**×**	**×**	✔ ^a^	✔
Fmoc‐Asp(OtBu)‐OH	✔ ^a^	✔	✔	✔	✔
Fmoc‐Gln(Trt)‐OH	**×**	**×**	✔ ^a^	✔	✔
Fmoc‐Gly‐OH	✔	✔	✔	✔	✔
Fmoc‐Ile‐OH	✔	✔	✔	✔	✔
Fmoc‐Leu‐OH	✔	✔	✔	✔	✔
Fmoc‐Phe‐OH	**×**	**×**	**×**	✔ ^a^	✔
Fmoc‐Tyr(tBu)‐OH	**×**	✔ ^a^	✔	✔	✔
Fmoc‐Val‐OH	✔	✔	✔	✔	✔

^a^ Only when the active ester is formed.

The most sterically hindered Fmoc‐AAs are not soluble in pure IPA; their solubility is enhanced by the addition of moderate percentages of DMSO, but in some cases [Fmoc‐Asn(Trt)‐OH, Fmoc‐Gln(Trt)‐OH, Fmoc‐Phe‐OH, Fmoc‐Tyr(tBu)‐OH] the complete solubilization is achieved only when the amino ester is formed. Although the initial idea was to use IPA as a pure solvent for the synthesis of peptide chains, the poor solubility of some amino acids, even in the form of active esters, forced us to opt for the use of DMSO/IPA mixtures, but selecting those with the lowest percentage of DMSO required to solubilize the reagents.

### 3.4 Synthesis of Model Peptides

This study was focused on two different model peptide sequences: Fmoc‐Leu‐Leu‐Val‐Phe‐NH_2_ and H‐Val‐Gln‐Ala‐Ala‐Ile‐Asp‐Tyr‐Ile‐Asn‐Gly‐NH_2_, which corresponds to the known ACP(65–74) sequence. The first sequence was chosen for its short length and hydrophobic nature, while ACP(65–74) is a well‐known [58], challenging sequence frequently used to validate new synthetic protocols.

Both sequences were synthesized using the same protocol, employing the Fmoc strategy in the set of solvents discussed above. In each synthesis, the resin was swollen for 30 min, and Fmoc removal was performed using a 20% piperidine solution. The carboxyl group was activated using Oxyma Pure/DIC, and the reaction was driven for 50 min. The washing step was maintained only after the Fmoc removal, avoiding washings after the coupling reaction [21]. This modification allowed us to reduce the volume of solvent waste associated with the entire synthetic protocol.

#### 3.4.1 Synthesis of Fmoc‐LLVF‐NH_2_

**FIGURE 3:**
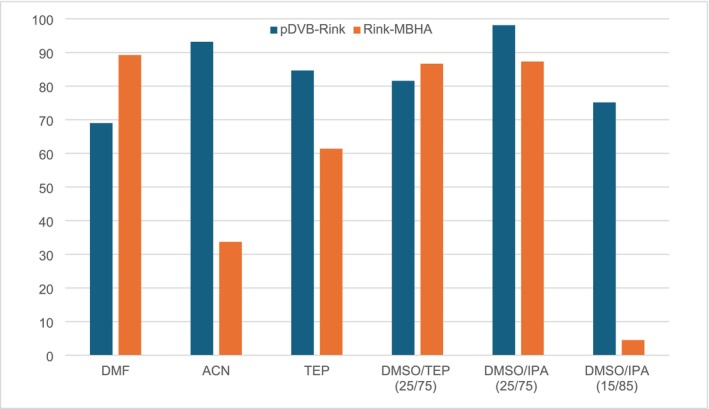
Purity of crude Fmoc‐LLVF‐NH_2_ synthesized on pDVB‐Rink and Fmoc‐Rink MBHA resins in different solvents.

Figure 3 reports the crude purities for Fmoc‐LLVF‐NH_2_ peptide in different experimental conditions.

**FIGURE 4:**
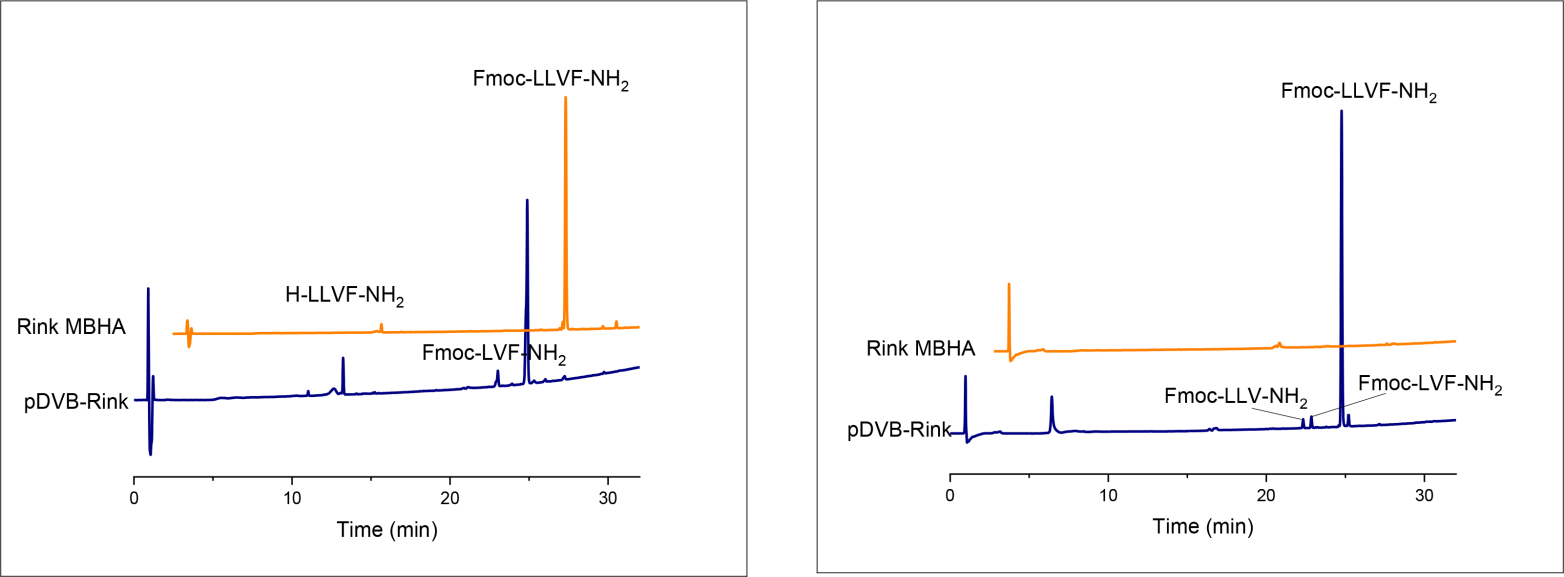
HPLC profiles of the model peptide Fmoc‐LLVF‐NH_2_, synthesized on different resins in DMF (left) and DMSO/IPA 15/85 v/v (right).

In standard conditions, using DMF as the solvent, the Fmoc‐Rink MBHA resin allowed us to obtain the target peptide in high purity (89%, Figure 4, left), and with an overall better performance as compared to the pDVB‐Rink resin. In the DMSO/TEP mixture, the results found for the two resins are similar (82% for pDVB‐Rink and 87% for Fmoc‐Rink MBHA resins, respectively) (see S.I.). However, in the other conditions tested, the purity of Fmoc‐LLVF‐NH_2_ synthesized on pDVB‐Rink was always higher, with values ranging from 75% (DMSO/IPA 15/85 v/v) to 98% (in DMSO/IPA 25/75 v/v). Also, it is worth noting that in DMSO/IPA (15/85 v/v), the synthesis on the conventional Rink MBHA resin resulted in an undetectable amount of the target peptide, whereas the synthesis on pDVB‐Rink yielded the desired product in good purity. Only minor by‐products were detected by HPLC‐MS (des‐Leu and des‐Phe, Figure 4, right).

The use of pure TEP as the solvent resulted in a better performance of the pDVB‐Rink resin characterized by a higher purity (85%) of Fmoc‐LLVF‐NH_2_. In spite of the modest sustainability of ACN, in this study, it was considered too as a solvent for the synthesis of peptides [26]. In this condition, the target product was obtained in high purity (93%) using pDVB‐Rink as the solid support, a result comparable to the best solvent considered in our work (DMSO/IPA 25/75 v/v) (see S.I.).

#### 3.4.2 Synthesis of ACP(65–74)

**FIGURE 5:**
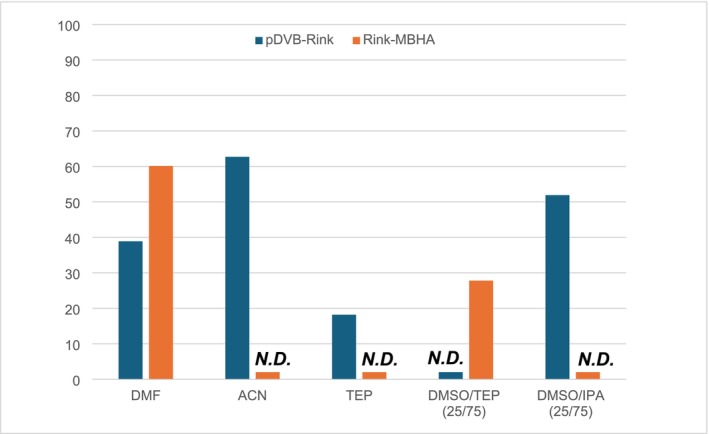
Comparison of the purity for ACP(65–74) synthesized on pDVB‐Rink and Fmoc‐Rink MBHA resins in different solvents (*N.D*. stands for the hardly detectable target compound).

ACP(65–74) is a well‐known, difficult sequence used to prove the effectiveness of new synthetic protocols. As the primary focus of this work was the preliminary screening of solvents alternative to DMF for the SPPS on the new pDVB‐Rink resin the synthetic protocol was not optimized. Figure 5 reports the crude purities of the target peptide prepared in different experimental conditions.

**FIGURE 6:**
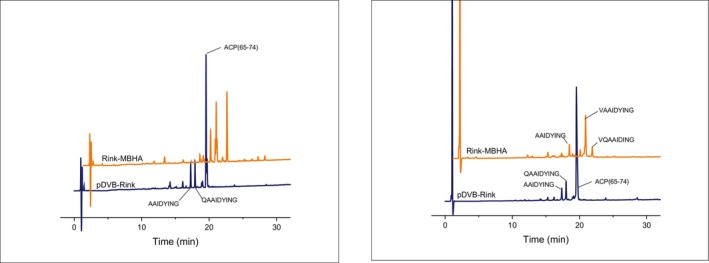
HPLC profiles of ACP(65–74), synthesized on different resins in DMSO/IPA 25/75 v/v (left) and ACN (right).

Again, under standard conditions in DMF, the crude purity is higher when using the commercial Rink MBHA resin as compared to the pDVB‐Rink resin (60% vs. 39%, respectively). The use of TEP and its mixture with DMSO resulted in poor crude purity (< 20%) for both resins. Interestingly, as observed for Fmoc‐LLVF‐NH_2_, in DMSO/IPA (25/75 v/v) and ACN the pDVB‐Rink resin performed much better than the commercial resin (Figure 6): The crude purity was 52% for the DMSO/IPA (25/75 v/v) mixture and 63% for ACN, while no product was detected with the commercial resin. Most of the by‐products of these syntheses are missing one or more amino acids. The most important are the products characterized by des‐Val, des‐Gln and the peptide in which both of these two amino acids are missing.

The nature of the main impurities confirms that the pDVB‐Rink resin shows a typical SPPS behavior and that classical strategies can be adopted to improve the purity of the crude (e.g., varying the reaction times and temperature). Furthermore, other approaches can be considered to enhance the sustainability of the process, such as evaluating alternative but equally or more sustainable activation methods [59, 60, 61, 62] and considering the use of microwave technology [63] to reduce reaction times.

To the best of our knowledge, this is the first successful application of highly crosslinked mesoporous pDVB‐Rink polymers, properly functionalized to serve as solid support in SPPS overcoming the typical limitations of common gel‐type resins with solvents different from DMF. This novel material expands the range of solvents that can be used in SPPS.

## 4 Conclusions

In this work, we reported the use of an innovative pDVB support functionalized with the Fmoc‐Rink amide linker and tested in the SPPS of the model peptides Fmoc‐LLVF‐NH_2_, a short and highly hydrophobic sequence, and ACP(65–74), a difficult sequence often used to validate new synthetic protocols. The most striking feature of the pDVB support is the stability of its expanded morphology in a variety of solvents, including those where standard polystyrene‐based resins fail. This feature allowed us to move away from the standard SPPS solvents DMF and NMP to more sustainable ones including TEP, ACN, and IPA/DMSO mixtures. To validate our results, the model peptides were synthesized in the same solvents using a commercial gel‐type resin, Rink Amide MBHA. In DMF, as far as the crude purity is concerned the latter generally performed better, but in the other solvents the results obtained for Fmoc‐LLVF‐NH_2_ are comparable (DMSO/TEP, DMSO/IPA‐25/75 v/v) or clearly better with the new pDVB‐Rink support (ACN, TEP, DMSO/IPA‐15/85 v/v). In the synthesis of ACP(65–74) with both supports the crude purities were lower in all the solvent tested. However, the new mesoporous pDVB‐Rink resin performed much better than the commercial gel‐type Rink‐MBHA resin in ACN and DMSO/IPA (15/85 v/v). Although obtained through non‐optimized synthetic protocols, these results are amenable to open interesting perspectives for greening SPPS, particularly as they show that a number of environmentally friendly solvents can be used. This study will be extended to other peptide sequences, the synthetic protocol will be optimized by working on more efficient activation methods and techniques, such as microwave technology.

## Conflicts of Interest

The authors declare no conflicts of interest.

## Supporting information


**Figure S1** Examples of the solubility of Fmoc‐AA in pure IPA and its solubility after formation of the active ester.
**Figure S2** Solubility of Fmoc‐Phe‐OH and Fmoc‐Asn(Trt)‐OH in different conditions.
**Figure S3** Solubility of Fmoc‐Gln(Trt)‐OH in different conditions.
**Figure S**4 Solubility of Fmoc‐Gln(Trt)‐OH in different conditions.
**Figure S5** Swelling process of the pDVB‐Rink resin tested in this work.
**Figure S6** Swelling process of the Rink‐MBHA resin tested in this work.
**Figure S7** HPLC profiles of the model peptide Fmoc‐LLVF‐NH2 synthesized on different resins in different solvents.
**Figure S8** MS analysis of Fmoc‐LLVF‐NH2 peptide products.
**Figure S9** HPLC profiles of ACP(65–74) synthesized on different resins in DMF.
**Figure S10** MS analysis of ACP(65–74) peptide products.

## Data Availability

The data that support the findings of this study are available on request from the corresponding author. The data are not publicly available due to privacy or ethical restrictions.
