# Supplementary material for: Toward a Green SPPS: The Use of an Innovative Mesoporous pDVB Support for Environmentally Friendly Solvents
Source: J Pept Sci. 2025 Jun 30;31(8):e70038. doi: 10.1002/psc.70038 (PMC12209690; doi:10.1002/psc.70038)
Supplement: Supplementary file 1 — Figure S1 Examples of the solubility of Fmoc‐AA in pure IPA and its solubility after formation of the active ester. Figure S2 Solubility of Fmoc‐Phe‐OH and Fmoc‐Asn(Trt)‐OH in different conditions. Figure S3 Solubility of Fmoc‐Gln(Trt)‐OH in different conditions. Figure S4 Solubility of Fmoc‐Gln(Trt)‐OH in different conditions. Figure S5 Swelling process of the pDVB‐Rink resin tested in this work. Figure S6 Swelling process of the Rink‐MBHA resin tested in this work. Figure S7 HPLC profiles of the model peptide Fmoc‐LLVF‐NH2 synthesized on different resins in different solvents. Figure S8 MS analysis of Fmoc‐LLVF‐NH2 peptide products. Figure S9 HPLC profiles of ACP(65–74) synthesized on different resins in DMF. Figure S10 MS analysis of ACP(65–74) peptide products. [file PSC-31-e70038-s001.pdf]

Toward a green SPPS: the use of an innovative mesoporous pDVB support for environmentally friendly solvents

Luana Lastella[1], Marco Zecca[1], Paolo Centomo[1], Karel Jeřábek[2], Fernando Formaggio[1,3], Ivan Guryanov[4,5], Antonio Ricci[5], Barbara Biondi[3]*

[1] Department of Chemical Sciences, University of Padova, 35131 Padova, Italy

[2] Institute of Chemical Process Fundamentals of the CAS, v.v.i., 16502 Prague 6, Czech Republic

[3] Institute of Biomolecular Chemistry, Padova Unit, 35131 Padova, Italy

[4] Institute of Chemistry, St. Petersburg State University, St. Petersburg 198504, Russia

[5] Fresenius Kabi Ipsum, 45010 Villadose, Rovigo, Italy

Table of contents

1. **Ability to solubilize Fmoc-aa-OH………………………………………………….pag.2**
2. **Swelling capacity……………………………………………………………………pag.4**
3. ***Synthesis of Fmoc-LLVF-NH$_2$***……………………………………………………**....pag.5**
4. ***Synthesis of ACP(65-74)***………………………………………………………….**pag.8**

Ability to solubilize Fmoc-aa-OH

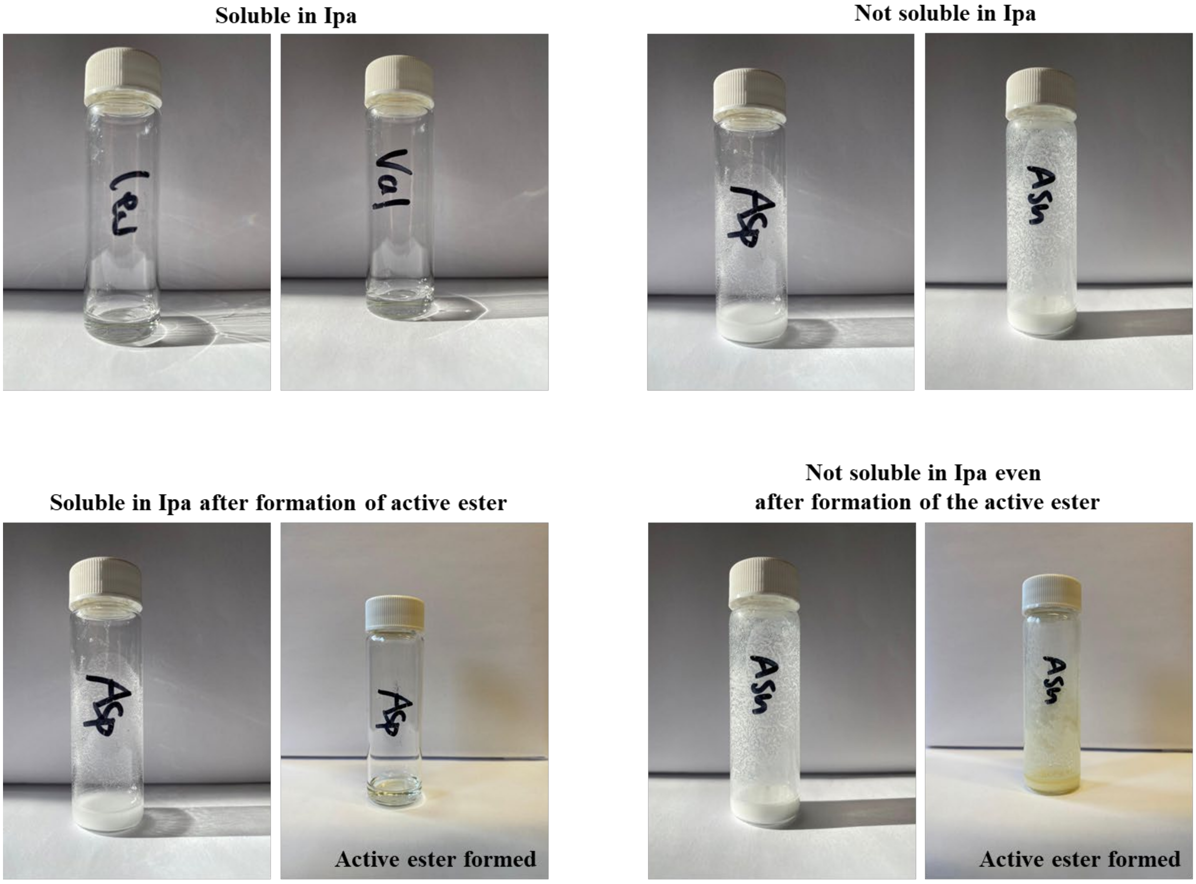

Figure SI-1. Examples of the solubility of Fmoc-AA in pure IPA and its solubility after formation of the active ester.

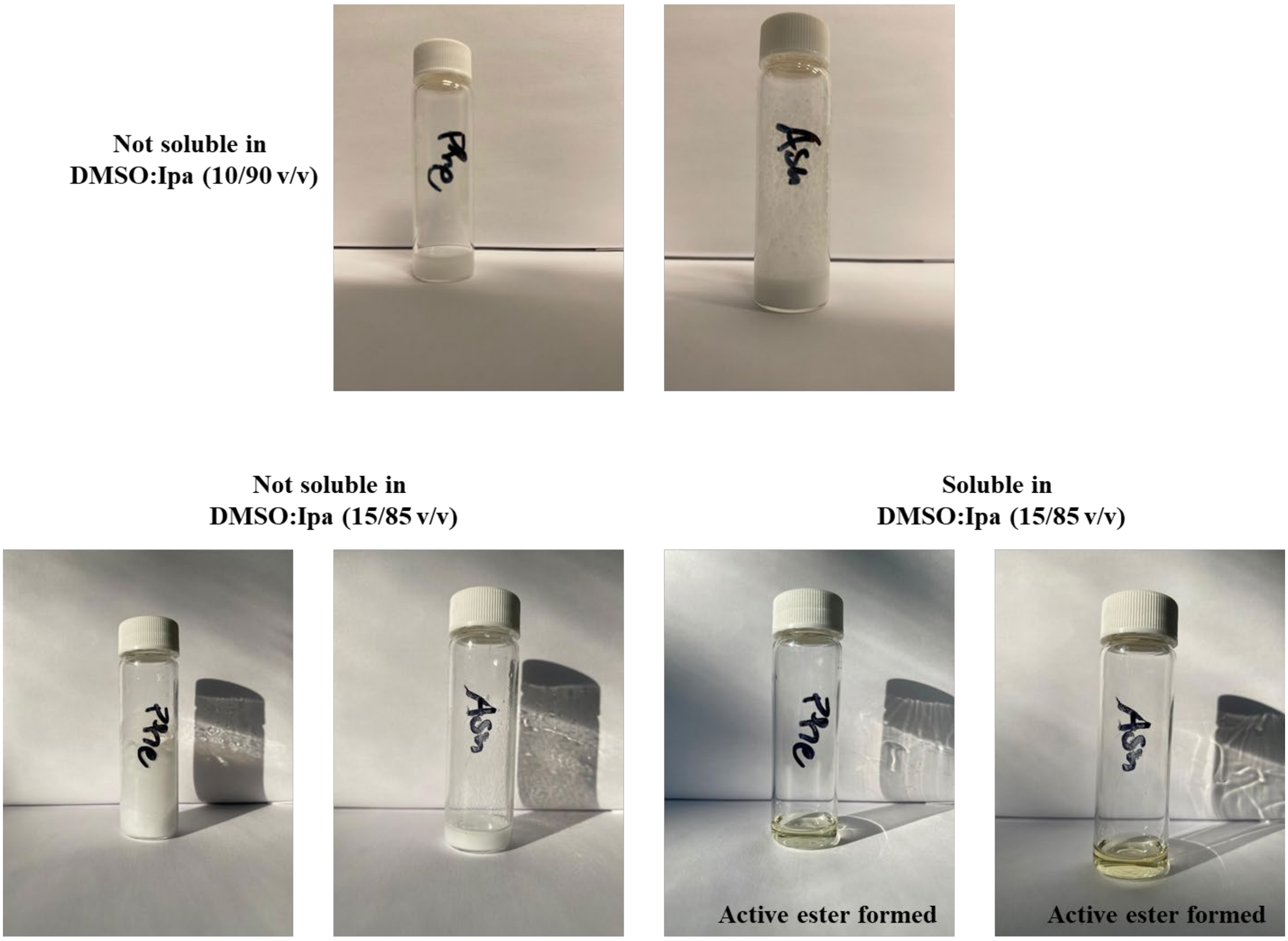

Figure SI-2. Solubility of Fmoc-Phe-OH and Fmoc-Asn(Trt)-OH in different conditions.

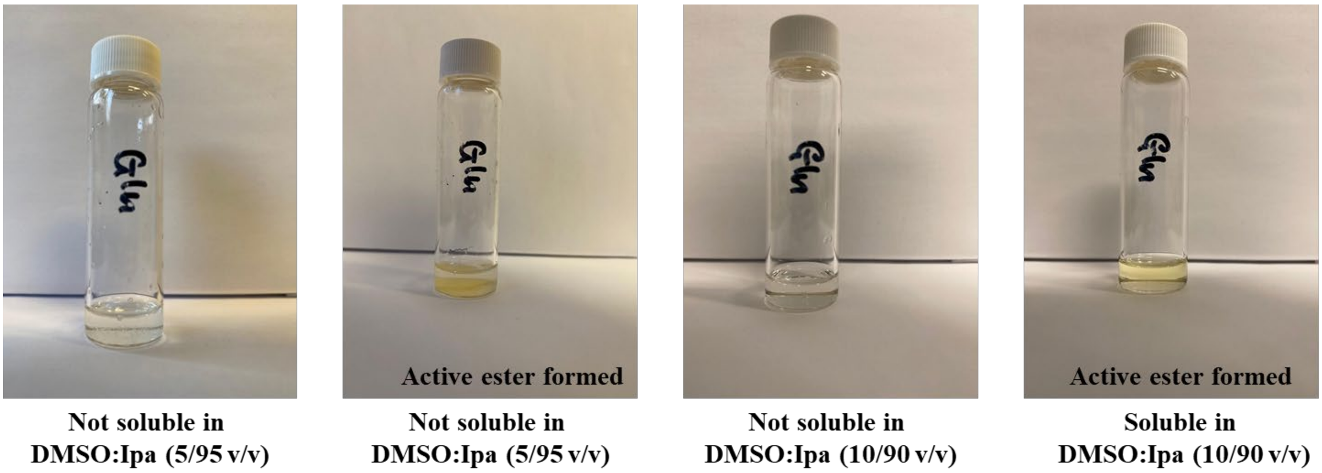

Figure SI-3. Solubility of Fmoc-Gln(Trt)-OH in different conditions.

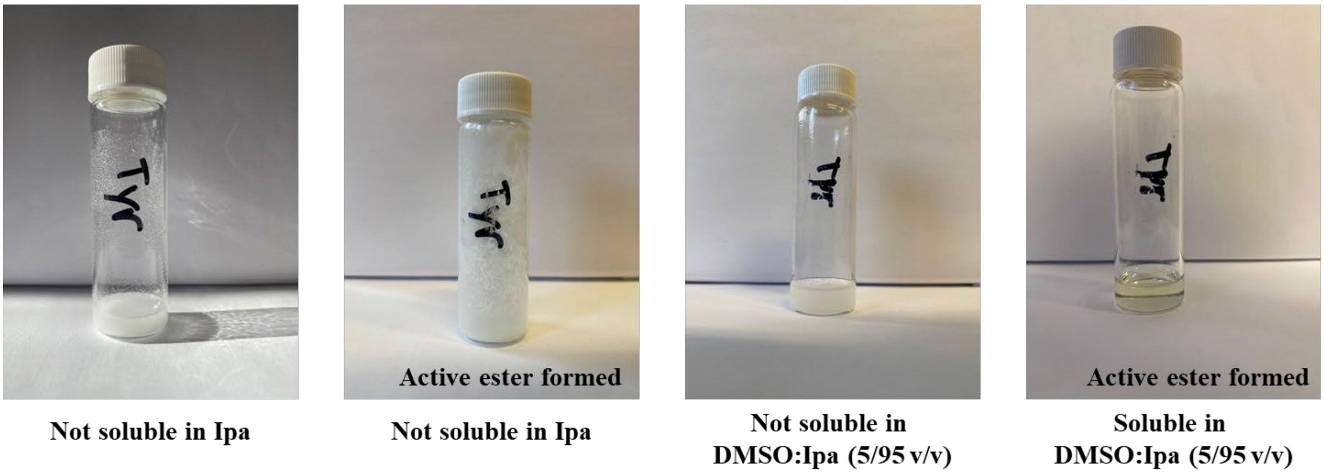

Figure SI-4. Solubility of Fmoc-Tyr(tBu)-OH in different conditions.

Swelling capacity

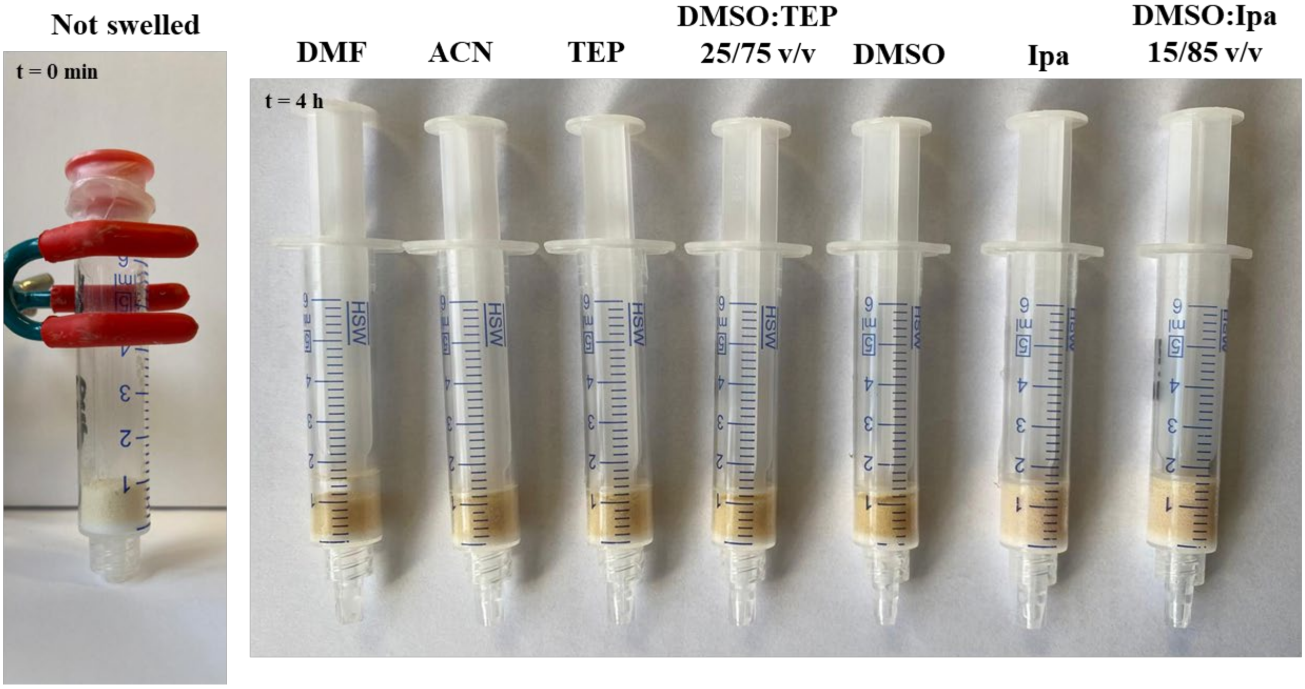

Figure SI-5. Swelling process of the pDVB-Rink resin tested in this work.

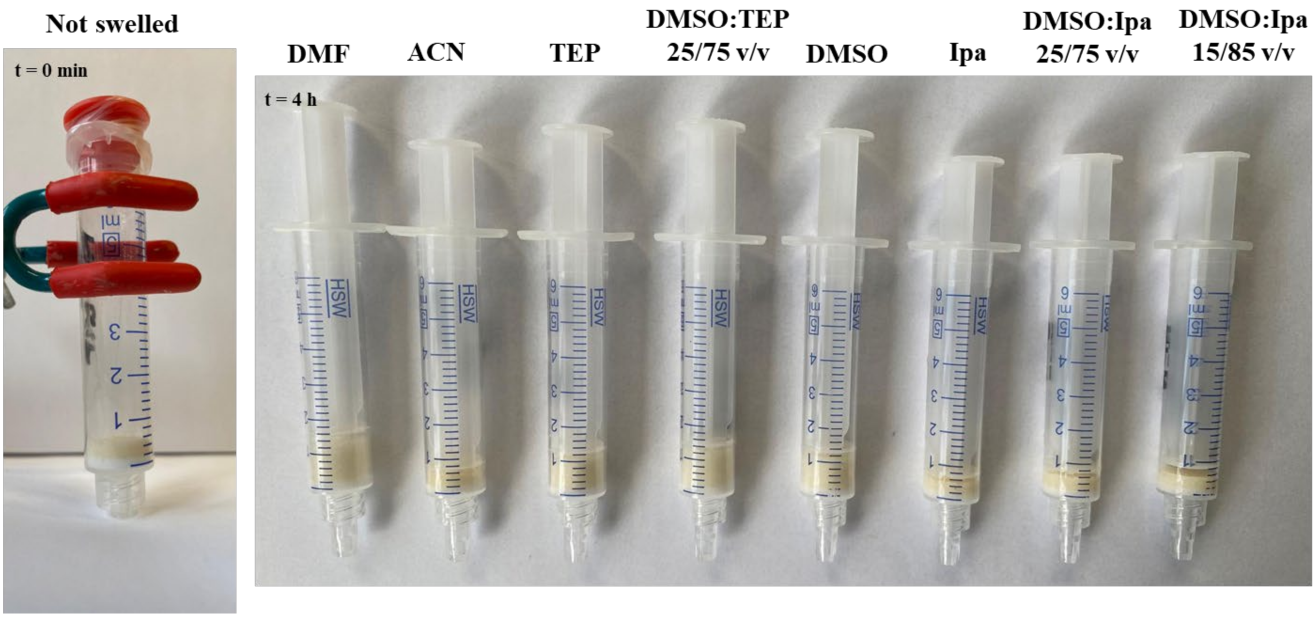

Figure SI-6. Swelling process of the Rink-MBHA resin tested in this work.

Synthesis of Fmoc-LLVF-NH₂

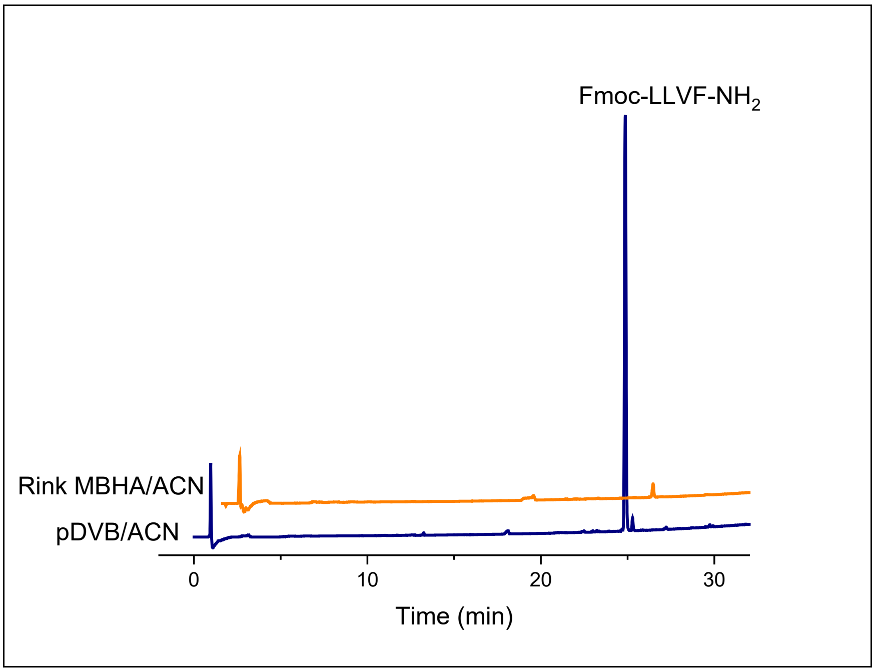

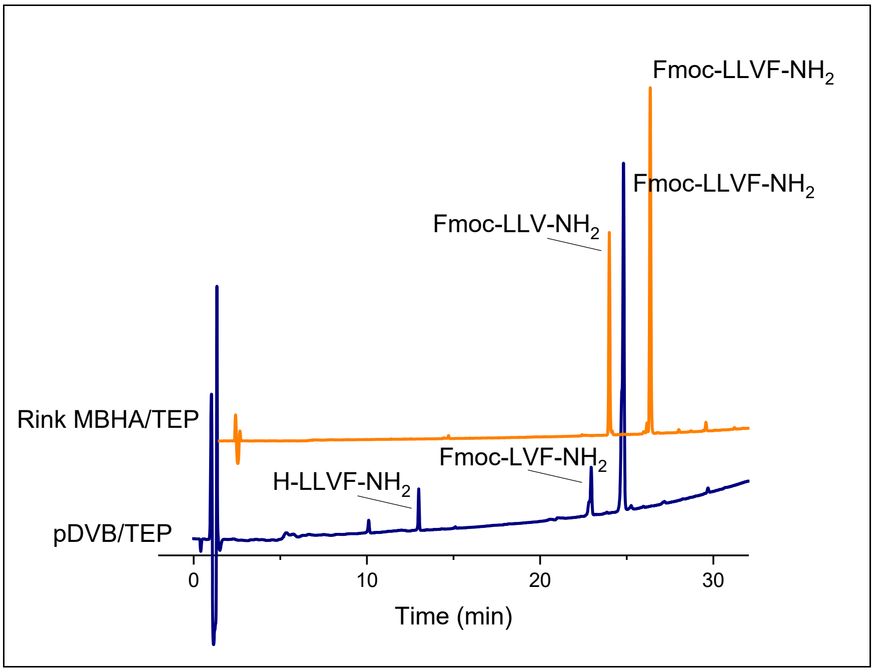

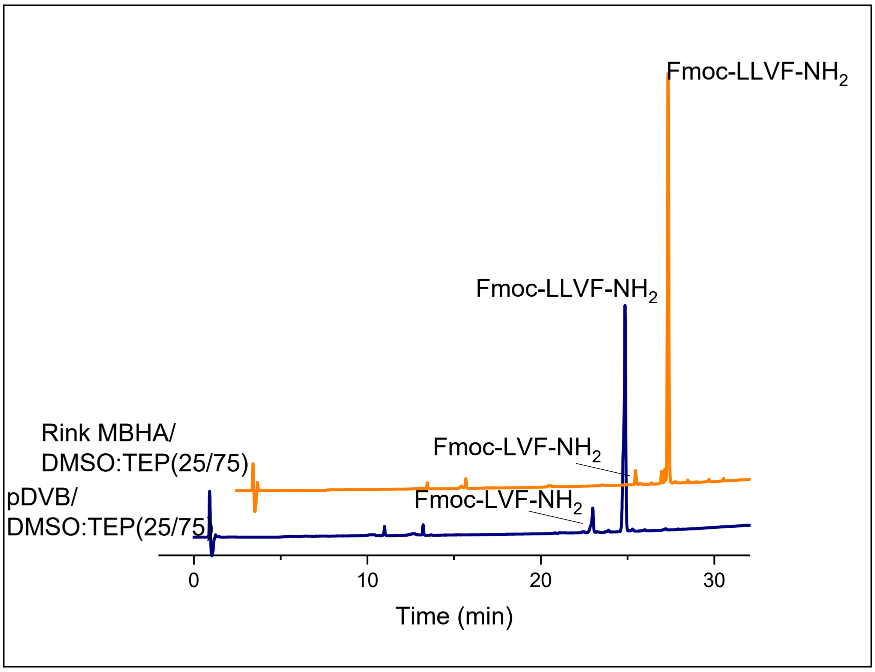

Figure SI-7. HPLC profiles of the model peptide Fmoc-LLVF-NH_2 synthesized on different resins in different solvents.

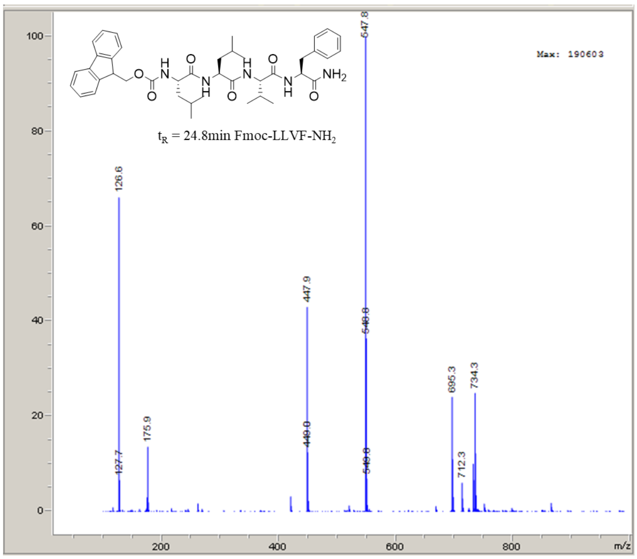

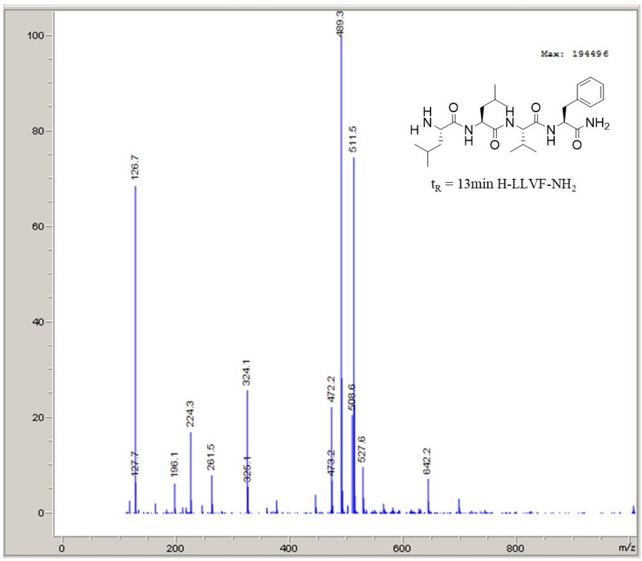

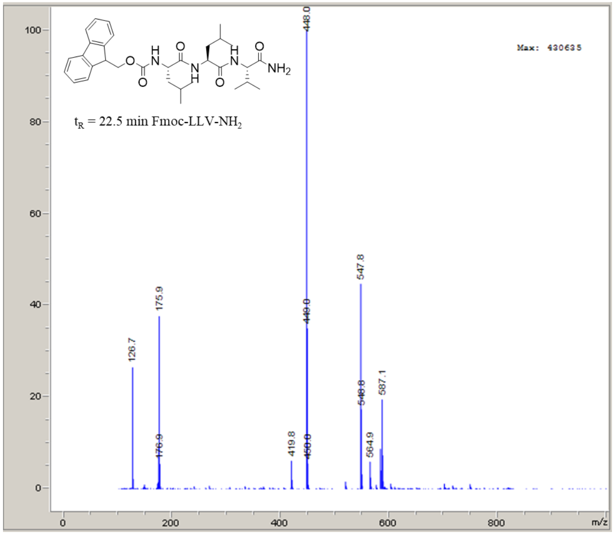

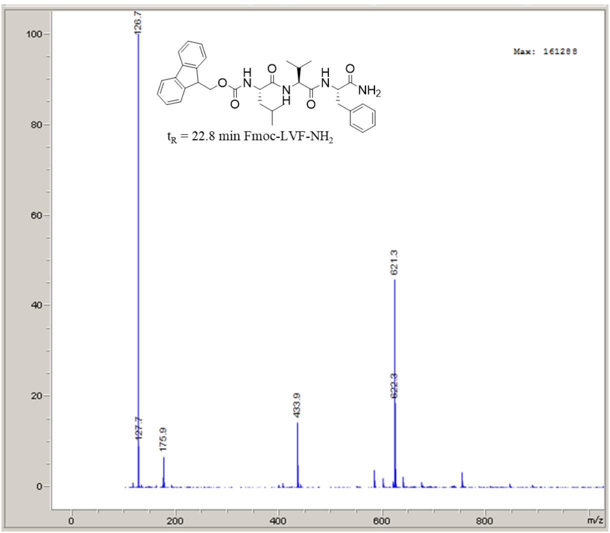

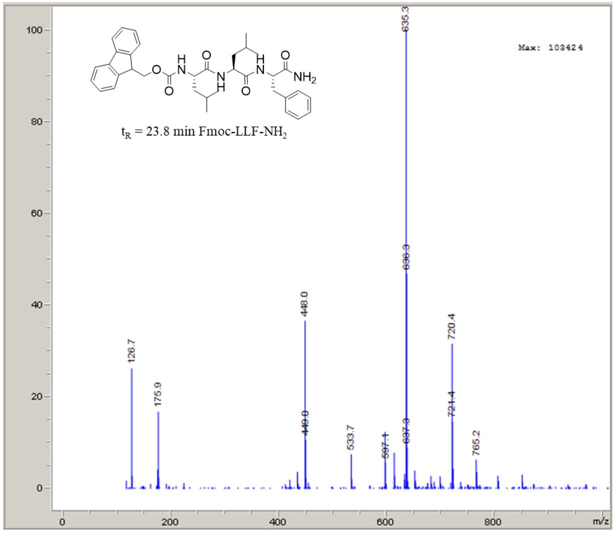

Figure SI-8. MS analysis of Fmoc-LLVF-NH_2 peptide products.

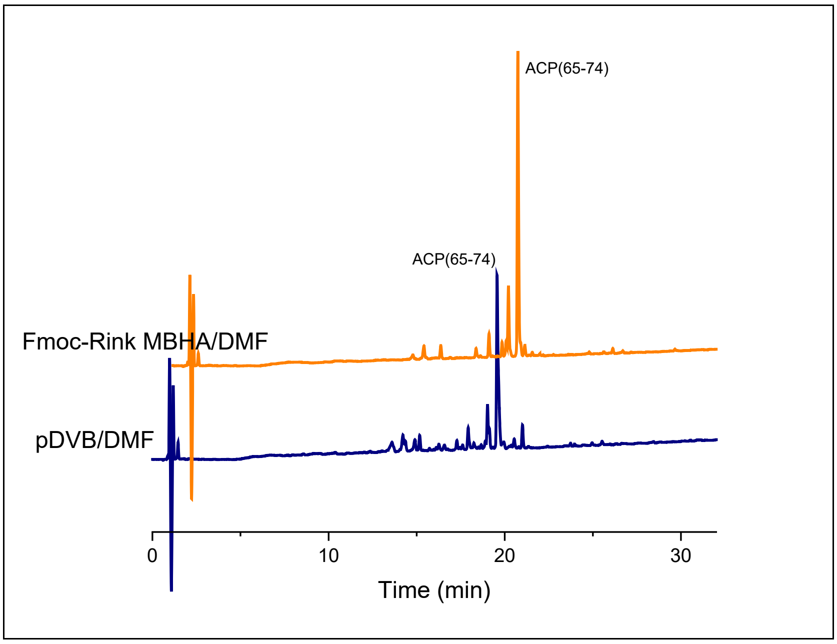

Figure SI-9. HPLC profiles of ACP(65-74) synthesized on different resins in DMF.

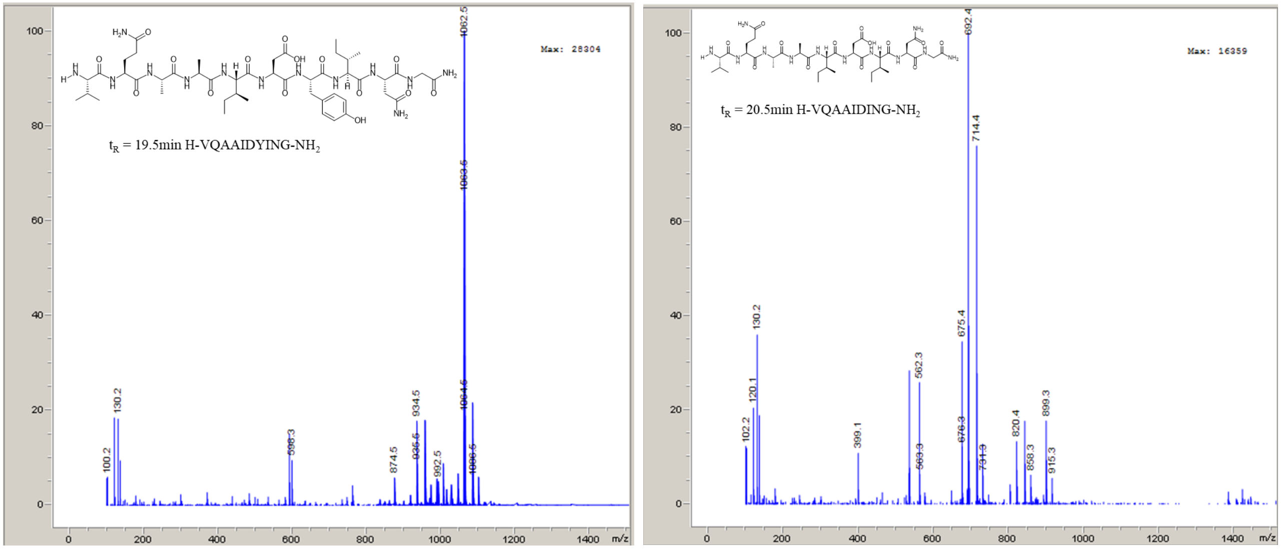

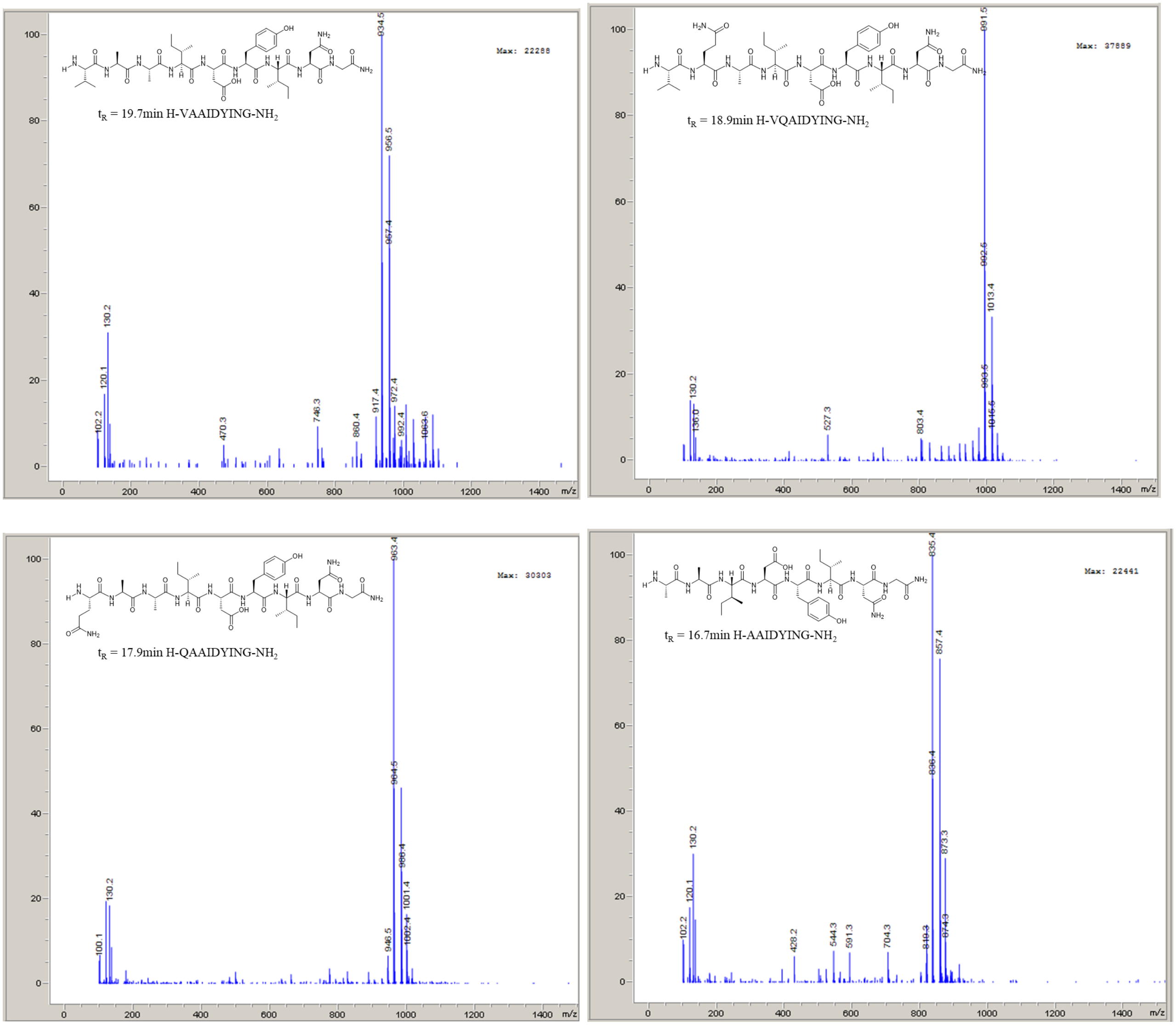

Figure SI-10. MS analysis of ACP(65-74) peptide products.
